# Structural and Functional Reorganization Within Cognitive Control Network Associated With Protection of Executive Function in Patients With Unilateral Frontal Gliomas

**DOI:** 10.3389/fonc.2020.00794

**Published:** 2020-05-27

**Authors:** Yong Liu, Guanjie Hu, Yun Yu, Zijuan Jiang, Kun Yang, Xinhua Hu, Zonghong Li, Dongming Liu, Yuanjie Zou, Hongyi Liu, Jiu Chen

**Affiliations:** ^1^Department of Neurosurgery, The Affiliated Brain Hospital of Nanjing Medical University, Nanjing, China; ^2^Institute of Brain Functional Imaging, Nanjing Medical University, Nanjing, China; ^3^Institute of Neuropsychiatry, The Affiliated Brain Hospital of Nanjing Medical University, Nanjing, China; ^4^School of Biomedical Engineering and Informatics, Nanjing Medical University, Nanjing, China; ^5^Department of Radiology, The Affiliated Brain Hospital of Nanjing Medical University, Nanjing, China

**Keywords:** cognitive control network, compensation hub, executive function, fMRI, frontal gliomas

## Abstract

**Background:** The cognitive control network (CCN) is widely considered to be a frontoparietal circuit that is involved in executive function. This study aimed to investigate the structural and functional plasticity within the CCN in unilateral frontal gliomas, which are associated with the protection of executive functions.

**Methods:** To detect structural and functional changes within the CCN, we measured gray matter (GM) volume, regional homogeneity, the amplitude of low-frequency fluctuation (ALFF), degree centrality, and functional connectivity within the CCN in 37 patients with gliomas invading the left frontal lobe (*n* = 16) or the right frontal lobe (*n* = 21) and 40 healthy controls (CNs). Partial correlation analysis was performed to assess the association between the altered structural and functional indices and executive function.

**Results:** When the tumor invaded the left frontal lobe, the patients showed reduced ALFF in the dorsal medial prefrontal cortex (dmPFC) within the CCN and increased ALFF in the right superior parietal cortex (rSP) within the CCN compared to the CNs. When the tumor invaded the right frontal lobe, the patients showed significantly increased GM volume and ALFF in the left superior parietal cortex (lSP) within the CCN compared to the CNs. Furthermore, the patients showed significantly increased functional connectivities between the lSP and the dmPFC and between the lSP and the rSP within the CCN compared to the CNs. Increased ALFF in the lSP within the CCN was positively correlated with executive function.

**Conclusions:** Tumors invading the frontal lobe induced contralesional structural and functional reorganization within the posterior CCN in patients with unilateral frontal gliomas. This further suggests that the contralesional superior parietal cortex acts as a functional compensation hub within the CCN, which may protect it against the detrimental effects of tumor invasion on executive functions.

## Introduction

Gliomas are considered to be the most common primary brain tumors (1), with the highest proportion of frontal lobe invasion (2). To our knowledge, local frontal lesions, which are located in the cognitive control network (CCN) involved in executive function, can affect the executive function of the brain (3–7). Interestingly, clinical observations show that patients with frontal gliomas retain intact executive function. Numerous studies have consistently indicated that this local lesion may induce brain neuroplasticity that is an intrinsic adaptive property of the CNS associated with the retention of cognition (8). However, little is known about whether tumor invasion induces functional or morphological remodeling in patients with frontal gliomas, contributing to the clinical compensation phenomenon of intact executive function. Thus, it is important to facilitate our understanding of compensatory mechanisms to improve comprehensive preoperative planning and the development of neurorehabilitation strategies in patients with frontal gliomas.

Growing evidence suggests that brain plasticity can be attributed to the functional or morphological remodeling of the neural organization of the CNS (9, 14), which is associated with the maintenance of cognitive function (10–12). A large number of structural and functional neuroimaging studies have identified four patterns of brain remodeling: perilesional recruitment, contralateral homotopic enrollment, ipsihemispheric remote recruitment, and contrahemispheric remote recruitment (13–16). Some studies have emphasized that it may be better to assess the effect of tumor invasion over the whole brain network involved in contralateral remote areas of the lesioned hemisphere (17–19) than in isolated brain regions (20–22). To date, however, it is unclear whether a functional and morphological reorganization of the cortex takes on a compensatory role in the contralesional remote areas within the lesioned hemisphere in a network-based form. Here, we specifically focused on the involvement of contralateral remote areas in the lesioned area within the CCN in lesion-induced structural and functional neuroplasticity in the maintenance of cognitive functions.

The CCN is a frontoparietal circuit comprising the main brain regions in the dorsal medial prefrontal cortex, bilateral prefrontal cortex, and bilateral superior parietal cortex (5, 23–26), involved in top-down, attention-dependent executive functions, such as decision-making and task-switching (27–29). Several functional MRI (fMRI) studies have suggested that lesions of a certain brain region within the network (such as the language network) can induce functional reorganization within the network (17–19, 30, 31). Specifically, a recent study reported that functional reorganization of the CCN may mitigate the detrimental effects of white matter lesions on executive functions in the elderly (10). Most importantly, an fMRI study suggested that lesions involving high-centrality nodes (hubs) within the networks induced larger and more widespread functional connectivity effects (32). Thus, based on the above-mentioned studies, we hypothesized that lesions invading the frontal lobe would induce structural and functional reorganization of contralesional remote areas within the CCN in patients with unilateral frontal gliomas. We further speculated that there was a functional compensation hub within the CCN that would protect against the detrimental effects of tumors invading executive function in patients with unilateral frontal gliomas.

## Methods and Materials

### Subjects

This study recruited a total of 37 patients with frontal gliomas invading the left frontal lobe (FronL group, *n* = 16, mean age 45.44 ± 12.12 [18-79] years, sex ratio 8 women/8 men) or the right frontal lobe (FronR group, *n* = 21, mean age 46.90 ± 15.46 [22-60] years, sex ratio 7 women/14 men) in the Department of Neurosurgery at the Affiliated Brain Hospital of Nanjing Medical University. Of the 37 patients with frontal glioma, according to the World Health Organization (WHO) classification criteria, 15 patients (9 patients from MRI protocol 1, and 6 patients from MRI protocol 2) exhibited low-grade glioma (LGG) (WHO I or II), and 22 patients (7 patients from MRI protocol 1, and 15 patients from MRI protocol 2) exhibited high-grade glioma (HGG) (WHO III or IV). Furthermore, The mean tumor volume is 103.26 ± 77.53 (43.85–126.56) cm^3^ for patients with left frontal gliomas, and 101.58 ± 65.78 (46.75–132.06) cm^3^ for patients with left frontal gliomas. The mean total intracranial volume is 1505.25 ± 114.38 (1323.76–1580.28) cm^3^ for patients with left frontal gliomas, and 1521.65 ± 126.70 (1348.36–1663.27) cm^3^ for patients with left frontal gliomas. A neuropathologist determined the WHO grade of the gliomas from tumor tissue obtained during the surgical resection. A neuroradiologist and a neurosurgeon work together to determine the localization of the tumor using preoperative T1-, T2-weighted, and T1-contrasted images. To account for the potential effect in the general linear model (GLM) in this study, the tumor volume, total intracranial volume, age, gender, and education level were included as covariates (see below). Table 1 shows the patients' clinicopathological characteristics.

**Table 1:** Demographics and cognitive measures of the patients with frontal glioma and control subjects.

**Items**	**CN (*n* = 40)**	**All patients (*n* = 37)**	**FrontL (*n* = 16)**	**FrontR (*n* = 21)**
Age (years)	61.53 (7.40)	46.27 (13.95)^a^	45.44 (12.12)^a^	46.90 (15.46)^a^
Gender (M/F), *n*	18/22	22/15	8/8	14/7
Education level (years)	12.34 (2.37)	7.65 (4.01)^a^	7.88 (2.87)^a^	7.48 (4.89)^a^
Handedness	R	R	R	R
MRI protocol, type 1/type 2, n	40/0	16/21	7/9	9/12
Tumor volume, cm^3^	NA	102.42 ± 71.66 (43.85–132.06)	103.26 ± 77.53 (43.85–126.56)	101.58 ± 65.78 (46.75–132.06)
Total intracranial volume, cm^3^	1483.82 ± 126.26 (1302.36–1572.52)	1513.45 ± 120.54 (1323.76–1663.27)	1505.25 ± 114.38 (1323.76–1580.28)	1521.65 ± 126.70 (1348.36–1663.27)
Executive function				
Digit span test	7.95 (1.32)	7.32 (3.80)	8.33 (6.09)	6.85 (2.34)

DST, digit span test; FrontL, patients with left frontal glioma; FrontR, patients with right frontal glioma; NA, not available.

The values are expressed as the mean (standard deviation, SD).

a Significant differences were found between the CNs and patients with frontal glioma.

All p-values were obtained by t-tests, except for gender (chi-squared test). There are four illiterate glioma subjects. A total of eight subjects with right frontal glioma and 10 subjects with left frontal glioma did not complete the cognitive test. Due to a limited number of left frontal glioma patients with cognitive tests, we did not investigate the relationship between executive function and functional parameters in patients with left frontal glioma.

The inclusion criteria for the patient groups included: (1) tumor pathology confirmed as primary glioma by surgery, (2) tumor invasion that had not reached the central sulcus, (3) patients with unilateral tumor invasion, (4) no evidence of any shift of the midline structures (septa pellucidum, corpus callosum, third ventricle), (5) excluded brain injuries, and (6) impaired at the executive functions, which was indicated by a score of digit span test that was within ≤ 3 of age- and education-adjusted norms. The exclusion criteria were: (1) multiple lesion foci, (2) patients with a history of substance abuse, and (3) MRI contraindications. Based on the above criteria, 37 patients (mean age: 45.83 ± 13.13 years, 22 males and 15 females) were recruited for the study. Forty healthy control (CN) volunteers were recruited from normal community health screening, broadcasting station recruitment, and a newspaper advertisement. All subjects underwent a complete physical examination and executive function assessment (digit span test, see Table 1). Both all patients and CN are right-handed.

The study was approved by the responsible Human Participants Ethics Committee of the Affiliated Brain Hospital of Nanjing Medical University in Nanjing, China. Written informed consent was obtained from all participants.

### MRI Data Acquisition

We collected the preoperative MRI data of patients from 2013 to 2019. The MRI images were acquired before surgery with a 3.0 Tesla Verio Siemens scanner with an 8-channel head-coil in the Department of Radiology at the Affiliated Brain Hospital of Nanjing Medical University.

We used a 3D magnetization-prepared rapid gradient echo to obtain T1-weighted MR images with the following parameters: repeat time (TR) = 1,900 ms, echo time (TE) = 2.49 ms, time inversion (TI) = 900 ms, matrix = 256 × 256, flip angle (FA) = 90°, thickness = 1 mm, gap = 0.5 mm, and slices = 176.

Resting-state functional images for the patients were obtained in the same medical center with two sets of scan parameters. The second set of scan parameters were used to obtain MRI data for all of the CN subjects. We used a gradient-recalled echo-planar imaging sequence, including 140/240 volumes (for the first set of parameters between 2013 and 2016/the second set of parameters between 2017 and 2019, respectively) (33, 34), with repetition times (TR) = 2,000 ms/2,000 ms, echo times (TE) = 30 ms /30 ms, flip angles (FA) = 90°/90°, acquisition matrices = 64 × 64/64 × 64, fields of view (FOV) = 240 mm × 240 mm/220 mm × 220 mm, thicknesses = 3.0 mm/4.0 mm, gaps = 4 mm /0 mm, numbers of slices = 30/36, and voxel sizes = 3.75 × 3.75 × 4 mm^3^/3.4 × 3.4 × 4 mm^3^ to obtain the fMRI images.

Different parameters were used by our research team to optimize and improve the imaging protocol and were not related to the purposes of the study. Even if the parameters were homogeneous in the same scanner, parameter differences were taken into consideration in the GLM as a covariable of non-interest.

### Neuropsychological Assessments

All subjects included in this study were provided with unstructured clinical interview-based neuropsychological assessments which were performed by two experienced neuropsychologists to ensure the reliability of the results. Several classical neuropsychological tests including digit span test (DST), memory test, visuospatial test, math exam test, digital symbol substitution test, mapping test, and similarity test.

### Image Preprocessing

Conventional preprocessing steps were conducted using MATLAB2013b (http://www.mathworks.com/products/matlab/) and DPABI image processing software (35). The details of the image processing procedure are in our previously published study (35) and are provided in SI Methods S.1. In brief, the conventional preprocessing steps included removing the first ten images; slice-timing correction; motion correction (36); spatial normalization; nuisance covariate regression, including the Friston 24-parameter model (37, 38); and low-frequency band-pass filtering (0.01–0.1 Hz). Finally, we also performed spatial smoothing before calculating amplitude of low-frequency fluctuations (ALFF), degree centrality (DC), and seed-based functional connectivity, and after calculating regional homogeneity (ReHo).

### Definition of Seed Regions of Interest Within the CCN

We defined the precise locations of the seed regions of interests (ROIs) within the CCN based on previously published studies (5, 23–26). Then, we selected five ROIs based on *a priori* knowledge of the CCN presentation. These included the dorsal medial prefrontal cortex (dmPFC), the left anterior PFC (laPFC), the right anterior PFC (raPFC), the left superior parietal gyrus (lSP), and the right superior parietal gyrus (rSP).

### Amplitude of Low-Frequency Fluctuations (ALFF) Analysis

To characterize regional functional alterations in the patients, we calculated the regional ALFF. The details of the ALFF analysis procedure are in our previously published study (39) and are provided in SI Methods S.2.

### Regional Homogeneity (ReHo) Analysis

To characterize the similarity or homogeneity of the time series in a local neighborhood of voxels, we measured the ReHo. The details of the ReHo analysis procedure are in our previously published study (40) and are provided in SI Methods S.3.

### Degree Centrality (DC) Analysis

To characterize the node characteristics of large-scale brain intrinsic connectivity networks, we calculated DC, representing the number of direct connections for a given voxel in the voxel-based graphs (41, 42). The details of the DC analysis procedure are in our previously published studies (41, 42) and are provided in SI Methods S.4.

### Functional Connectivity Analyses

We extracted the individual averaged time courses for each region of the five ROI regions (dmPFC, laPFC, laPFC, lSP, and rSP) within the CCN (5, 23–25) separately as the reference time course and then calculated Pearson's correlation coefficients (*r*) between the averaged time courses of each seed pair. Finally, we performed Fisher's z-transformation to improve the normality of the correlation coefficients.

### Structural MRI Analysis

Structural MRI analysis was conducted with Statistical Parametric Mapping (SPM12, Wellcome Trust Center for NeuroImaging, University College, London, UK. available at: http://www.fil.ion.ucl.ac.uk/spm). In image preprocessing, firstly, structural images were manually reoriented and shifted to define the anterior commissure as the origin (mm coordinate 0, 0, 0). Secondly, we used the Diffeomorphic Anatomical Registration Through Exponentiated Lie Algebra (DARTEL) technique to normalize and segment the structural images into gray matter (GM), white matter (WM), and CSF (13). The native and DARTEL versions were provided for GM and WM tissues, and only native space was imported for CSF tissues (to compute intracranial volumes). Then, we used linear affine registration and non-linear deformation to normalize the individual GM and WM segmentations to the Montreal Neurological Institute (MNI) standard space. Then, we also modulated the GM and WM images to preserve the relative volume and correct for brain size. Finally, we resampled the GM and WM images to 3-mm cubic voxel resolutions and smoothed them using a 6-mm full width at half maximum.

### Statistical Analysis

Two-sample two-tailed *t*-tests and chi-squared tests were performed to compare the differences in demographic data and executive function between the CNs and patients (FrontL or FrontR).

We also used two-sample *t*-tests to compare the differences in the regional mean DC, ReHo, GM, ALFF maps, and FC matrix within the CCN between the CNs and the patients (FrontL or FrontR) after controlling the effects of demographic data (age, sex, and education level), parameter differences of MRI protocol, global intracranial volume, and tumor volume. For the ALFF, ReHo, GM, and DC results, all results were considered statistically significant at *p* < 0.05 (threshold-free cluster enhancement family-wise error, TFCE-FWE corrected) and voxels >30. The FC results were considered statistically significant at *p* < 0.05 (FDR correction for multiple comparisons). Plots and mean cross-correlation matrices between the groups were used to display the correlations between any ROI pair of the CCN.

Moreover, we performed a partial correlation analysis to investigate the association between executive function and the altered ALFF, ReHo, GM, DC, and FC parameters after controlling the effects of demographic data (age, sex, and education level), parameter differences of MRI protocol, global intracranial volume, and tumor volume. All results were considered statistically significant at *p* < 0.05.

## Results

### Demographic and Neuropsychological Characteristics

As shown in Table 1, no significant differences in gender or education level were observed between the FronL group or FronR group and the CN group (all *p* > 0.05). Patients in both the FronL group and the FronR group were younger age than the CN subjects (45.44 ± 11.11 vs. 61.53 ± 7.40 for the FronL group and 46.90 ± 15.46 vs. 61.53 ± 7.40 for the FronR group, all *p* < 0.05). Compared to the CN subjects, neither the FronL group nor the FronR group showed significant deficits in executive function (all *p* > 0.05).

### Comparison of Gray Matter (GM) Between the Frontal Patients and the CNs

When tumors invaded the left frontal lobe, the patients showed no significant differences in GM volumes in any brain regions (dmPFC, laPFC, laPFC, lSP, and rSP) within the CCN compared to the CNs (Figure S1).

**Figure 1:**
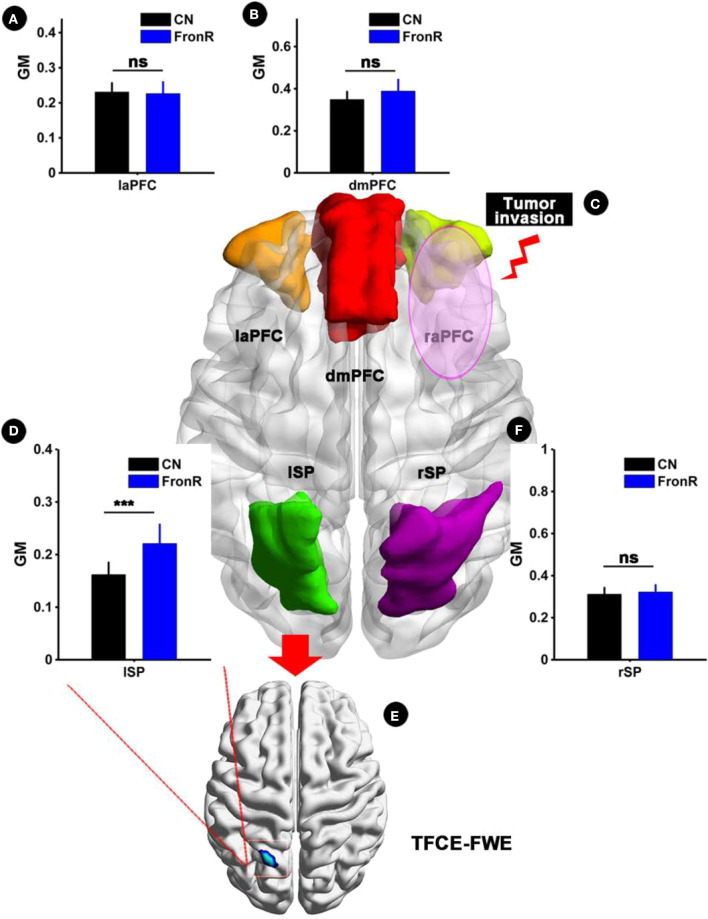
VBM analysis comparing FronR patients with CNs. GM volumes within the CCN in tumors invading the right frontal lobe derived from VBM analysis compared to CNs. The GM volumes are expressed in millimeters cubed and are presented as mean ± SD in **(A,B,D,F)**. **(C)** Shows five nodes within the CCN in a tumor invading the right frontal lobe. **(E)** Shows the brain regions of GM volume differences in the CCN in tumors invading the right frontal lobe compared to CNs. ***All results were thresholded at a voxel-wise *P* < 0.05 (threshold-free cluster enhancement family-wise error, TFCE FWE corrected) and cluster size >30 voxels; ns, not significant. DmPFC, dorsal mPFC (similar to superior medial frontal gyrus in AAL template); laPFC, left anterior PFC; raPFC, right anterior PFC; lSP, left superior parietal lobe; rSP, right superior parietal lobe; CN, controls; FrontR, patients with right frontal glioma; GM, gray matter.

Interestingly, when the tumor invaded the right frontal lobe, the patients displayed significantly increased GM volume in the lSP within the CCN compared to the CNs (*p* < 0.05, TFCE-FWE corrected and cluster size >30 voxels), as shown in Figure 1 and Table 2. No significant difference in GM volume was found in other regions within the CCN.

**Table 2:** Comparisons of gray matter (GM) and ALFF between frontal patients and CN subjects.

**Brain region**	**Peak MNI coordinate**			**Peak *T*-value**	**Cluster size (mm^**3**^)**
	***x***	***y***	***z***		
**GM**					
**(1) CN vs. FronL**					
No					
**(2) CN vs. FronR**					
L Superior parietal gyrus	−24	−63	69	−5.996	837
**ALFF**					
**(1) CN vs. FronL**					
R Superior medial frontal gyrus	12	51	33	4.2527	891
R Superior parietal gyrus	18	−81	51	−6.493	4,914
**(2) CN vs. FronR**					
L Superior parietal gyrus	−18	−84	48	−6.54	8,046

*CN, controls; FrontL, patients with left frontal glioma; FrontR, patients with right frontal glioma; GM, gray matter; ALFF, amplitude of low frequency fluctuation; L, left; R, right. All results were thresholded at a voxel-wise P < 0.05 (threshold-free cluster enhancement family-wise error, TFCE FWE corrected) and cluster size >30 voxels*.

### Comparisons of ALFF, ReHo, and DC Between the Frontal Patients and the CNs

**Figure 2:**
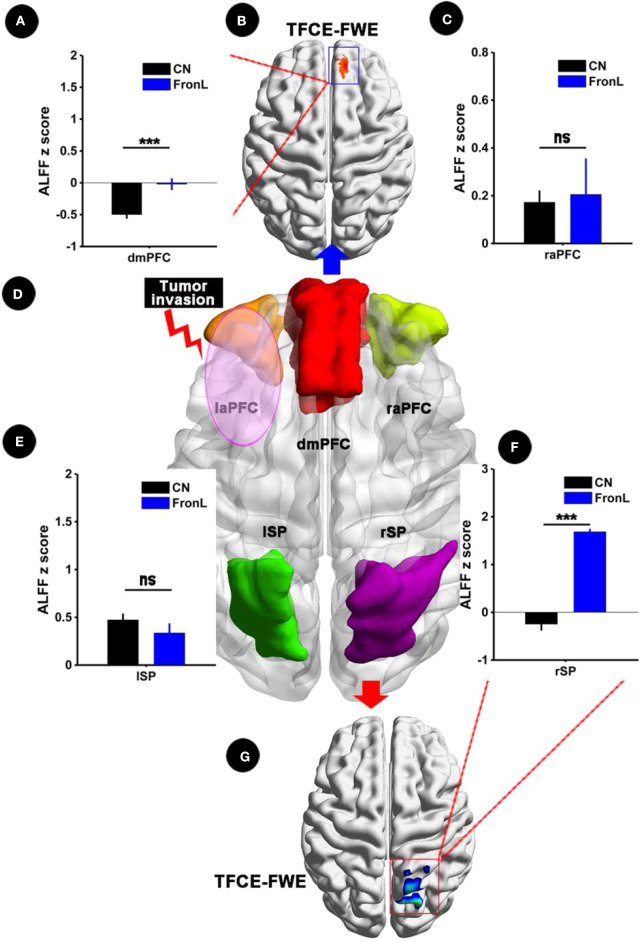
ALFF analysis comparing FronL patients with CNs. ALFF within the CCN in a tumor invading the left frontal lobe compared to a CN. ALFF z scoreS are presented as the mean ± SD in **(A,C,E,F)**. **(D)** Shows five nodes within the CCN in tumors invading the left frontal lobe. **(B,G)** Shows the brain regions of ALFF differences in the CCN in tumors invading the left frontal lobe compared to CNs. ***All results were thresholded at a voxel-wise *P* < 0.05 (threshold-free cluster enhancement family-wise error, TFCE FWE corrected) and cluster size >30 voxels; ns, not significant. DmPFC, dorsal mPFC (similar to superior medial frontal gyrus in AAL template); laPFC, left anterior PFC; raPFC, right anterior PFC; lSP, left superior parietal lobe; rSP, right superior parietal lobe; CN, controls; FrontL, patients with left frontal glioma; ALFF, amplitude of low frequency fluctuation.

When the tumor invaded the left frontal lobe, the patients showed significantly decreased ALFF in the dmPFC within the CCN and increased ALFF in the rSP within the CCN compared to the CNs (*p* < 0.05, TFCE-FWE corrected and cluster size > 30 voxels), as shown in Figure 2 and Table 2. No significant difference in ALFF was found in other regions within the CCN.

**Figure 3:**
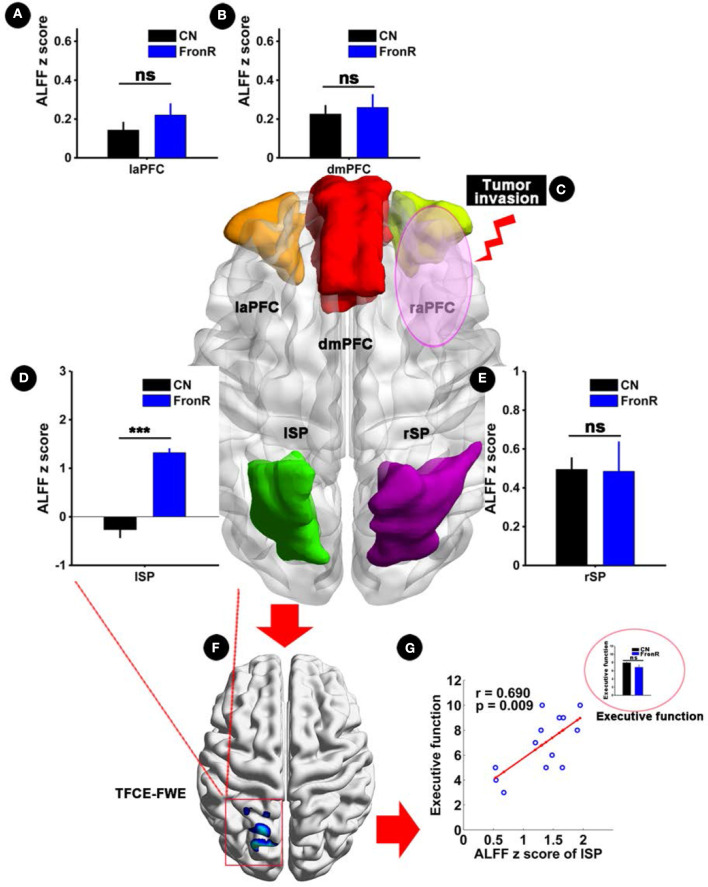
ALFF analysis comparing FronR patients with CNs and its relationship with executive function. ALFF within the CCN in tumors invading the right frontal lobe compared to CNs and its relationship with executive function. ALFF z scores are presented as mean ± SD in **(A,B,D,E)**. **(C)** Shows five nodes within the CCN of tumors invading the right frontal lobe. **(F)** Shows brain regions of ALFF differences in the CCN in tumors invading the right frontal lobe compared to CNs. **(G)** Shows the relationship between altered ALFF in the CCN in tumors invading the right frontal lobe and executive function. The small bar graph in **(G)** shows the comparison of executive function between the CN and FronR groups. ***All results were thresholded at a voxel-wise *P* < 0.05 (threshold-free cluster enhancement family-wise error, TFCE FWE corrected) and cluster size >30 voxels; ns, not significant. DmPFC, dorsal mPFC (similar to superior medial frontal gyrus in AAL template); laPFC, left anterior PFC; raPFC, right anterior PFC; lSP, left superior parietal lobe; rSP, right superior parietal lobe; CN, controls; FrontR, patients with right frontal glioma; ALFF, amplitude of low frequency fluctuation.

Interestingly, when tumors invaded the right frontal lobe, the patients exhibited significantly increased ALFF in the lSP within the CCN compared to the CNs (*p* < 0.05, TFCE-FWE corrected and cluster size >30 voxels), as shown in Figure 3 and Table 2. No significant difference in ALFF was found in other regions within the CCN.

When tumors invaded the left or right frontal lobe, the patients exhibited no significant differences in ReHo and DC in any brain region within the CCN compared to the CNs (Figure S2).

### Comparison of Functional Connectivity Within the CCN Between Frontal Patients and the CNs

When the tumor invaded the left frontal lobe, the patients showed no significant difference in functional connectivity within the CCN compared to the CNs.

**Figure 4:**
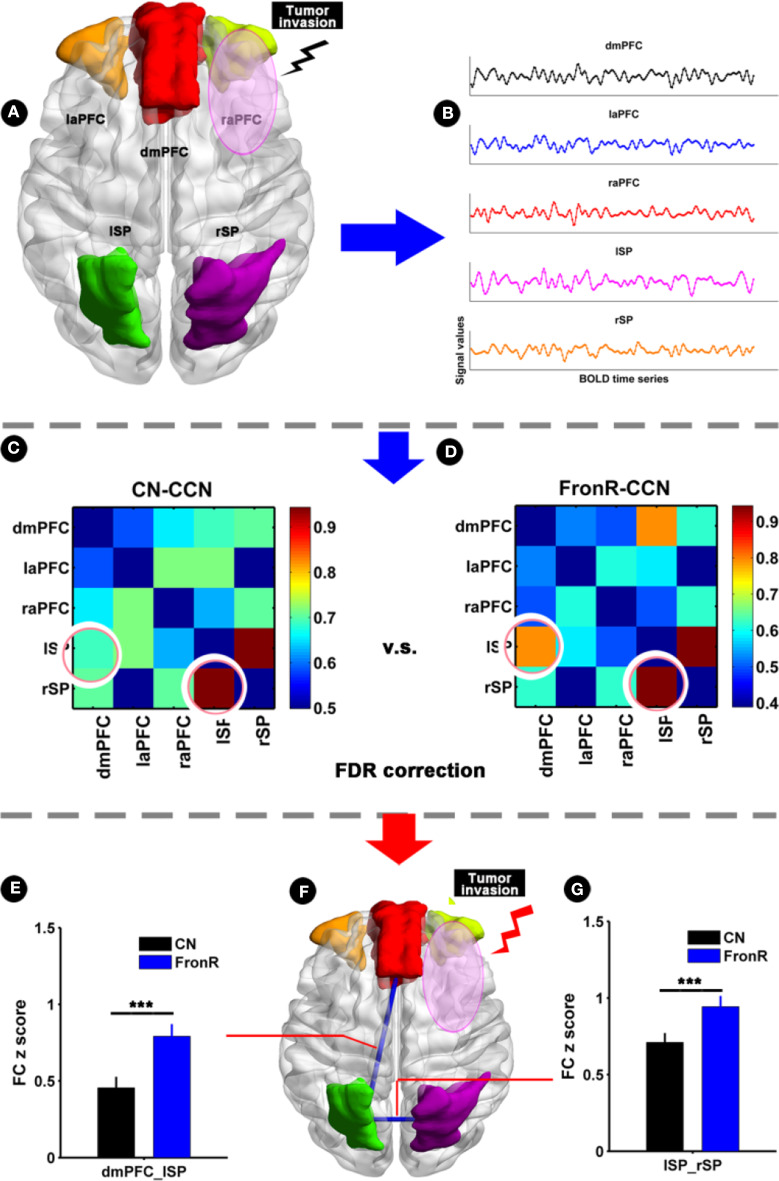
Functional connectivity within CCN comparing FronR patients with CNs. Histograms and FC matrices within the CCN in tumors invading the right frontal lobe compared to CNs. **(A)** Shows five nodes within the CCN in tumors invading the right frontal lobe. **(B)** Shows a schematic diagram of the BOLD time series extracted from five nodes within the CCN. **(C,D)** show the mean FC matrices within the CCN in the CN and FronR groups, respectively. The FC z scores of between-group comparison are presented as mean ± SD in **(E,G)**. **(F)** Shows altered FCs in the CCN of tumors invading the right frontal lobe compared to the CNs. ***FC results were considered statistically significant at *P* < 0.05 (FDR correction for multiple comparisons). DmPFC, dorsal mPFC (similar to superior medial frontal gyrus in AAL template); laPFC, left anterior PFC; raPFC, right anterior PFC; lSP, left superior parietal lobe; rSP, right superior parietal lobe; CN, controls; FrontR, patients with right frontal glioma; FC, functional connectivity; lSP-rSP, functional connectivity between lSP and rSP; dmPFC-lSP, functional connectivity between dorsal mPFC and lSP.

When the tumor invaded the right frontal lobe, the patients exhibited significantly increased functional connectivities between the lSP and the dmPFC and between the lSP and the rSP within the CCN compared to the CNs (*p* < 0.05, FDR corrected, Figure 4 and Table 2). There was no significant difference in paired connectivity between other regions within the CCN.

### Clinical Significance of Abnormal Structural and Functional Indices

Partial correlation analysis showed a significantly positive correlation between increased ALFF in the lSP within the CCN and executive function in patients with tumors invading the right frontal lobe (*r* = 0.690, *p* = 0.009), whereas no significant difference in executive function was found between the patients and the CNs (*p* > 0.05), as shown in Figure 3G. Furthermore, no significant correlations were found in the other indices (all *p* > 0.05).

## Discussion

To our knowledge, this study was the first to investigate whether lesions invading the frontal lobe would induce structural and functional reorganization of the contralesional remote areas within the CCN in patients with unilateral frontal gliomas, which are associated with the protection of executive functions. In particular, the most fascinating findings of this study should be emphasized. First, when the tumor invaded the left frontal lobe, the patients showed significantly decreased ALFF in the dmPFC within the CCN and increased ALFF in the rSP within the CCN compared to the CNs. When the tumor invaded the right frontal lobe, the patients showed significantly increased GM volume and ALFF in the lSP within the CCN compared to the CN. Furthermore, the patients showed significantly increased functional connectivities between the lSP and the dmPFC and between the lSP and the rSP within the CCN compared to the CNs. Finally, increased ALFF in the lSP within the CCN was positively correlated with executive function. Thus, the unique contribution of the present study was to identify the contralesional superior parietal cortex as a functional compensation hub within the CCN, which may protect against the detrimental effects of tumor invasion on executive function.

A strength of this work is that when the tumor invaded the right frontal lobe within the CCN, the patients showed significantly increased GM volume and ALFF in the lSP in the contralesional remote areas of the lesion region within the CCN. These findings suggest that the tumor invasion induced contralesional structural and functional reorganization within the CCN in patients with unilateral frontal gliomas and especially that the lSP plays a compensatory role in the CCN, which is mediated through the corpus callosum (12, 43). Therefore, our results further supported the fact that structural reorganization observed in patients with gliomas might be a physiologic basis for the high level of functional compensation (13). Indeed, several studies have speculated that structural reorganization may reflect the increases in cell size or spine density, as well as neural or glial cell genesis (44, 45). Furthermore, according to an adaptive mechanism hypothesis, fast-adjusting neuronal systems (46) and slow-evolving mechanisms (8) are considered to contribute to structural and functional compensation in patients with frontal gliomas. Interestingly, these results further corroborated our predictions that brain neuroplasticity was induced in the contralesional remote areas of the lesioned region within the CCN in patients with unilateral frontal gliomas. Some studies have indicated that brain reorganization due to tumor invasion is involved in distal regions relative to the lesion (9, 47), indicating that the effect of tumor invasion extends beyond the affected area. The explanation may be that the effect of tumor invasion comes into play in a network-based form rather than on the basis of the activity of isolated regions (20–22). In particular, previous studies have demonstrated the functional significance of distant interhemispheric communication (17–19). Furthermore, and most importantly, functional reorganization in the lSP within the CCN was associated with executive function. Therefore, these observations further suggest that functional reorganization may protect against the detrimental effects of tumor invasion on executive functions in patients with unilateral frontal gliomas.

Moreover, it is worth noting that when tumors invaded the left frontal lobe within the CCN, the patients only showed significantly increased ALFF in the rSP in the contralesional remote areas within the CCN compared to the CNs. Indeed, some functional neuroimaging studies have also suggested that lesions of a certain brain region within the network can induce functional reorganization within the network (17–19, 30, 31). However, this study did not find GM changes in this region. One reason for the findings could be that functional reorganization is already apparent when the structural reorganization is not very obvious at this stage. That is, structural reorganization requires a longer time than functional reorganization (48). Another reason for the findings could be that brain structural and functional reorganization may involve both compensatory and decompensatory processes as the disease progresses (49–51). Our patients with tumors invading the left frontal lobe could have been in a decompensated period. Further work will compare the differences in brain reorganization between low-grade glioma and high-grade gliomas to determine the difference between the compensatory and decompensatory periods.

The unique contribution of this study is that both structural and functional reorganizations converged in the contralesional superior parietal cortex of the tumor-invaded region within the CCN. These findings suggest that the superior parietal cortex may act as a functional compensation hub within the CCN. A large number of studies have consistently indicated that there are cortical hubs in functional networks, which may play crucial roles in moderating inter-regional neuronal communication (52, 53). In light of this framework, we speculated that cortical hubs will be first induced to activate the compensatory mechanism and then play a decompensatory role when the tumor invades the non-hub brain region. This hypothesis was supported and verified by our functional connectivity findings that showed significantly increased connectivities between the lSP and the dmPFC and between the lSP and the rSP within the CCN. Cortical lesions are thought to induce changes in the spontaneous coherence between close and distant regions within a functional network (54). Therefore, it is reasonable to speculate that the possible compensatory mechanism is that the functional compensation hub (i.e., the superior parietal cortex) may recruit other resources from functionally connected brain areas within the CCN to compensate for losses due to the detrimental effects of tumor invasion (21, 22, 24).

This study also had some limitations. Firstly, this study used a cross-sectional design, which could not observe longitudinal changes in the compensation hub. In the future, longitudinal studies will be needed to confirm the stability and persistence of the structural and functional reorganization of the contralesional remote areas in the lesioned areas within the CCN in patients with unilateral frontal gliomas. Secondly, this study only focused on the CCN. Other networks, such as the memory network, should be assessed in future studies. Some studies have indicated that frontal lobe lesions may be associated with impaired memory (55). Further work needs to confirm whether this pattern of structural and functional plasticity can extend to other networks. Thirdly, we used MRI data from different resting state functional MRI protocols. Different parameters were used by our research team to optimize and improve the imaging protocol and were not related to the purposes of the study. Even if the parameters were homogeneous in the same scanner, parameter differences were taken into consideration in the GLM as a covariable of non-interest. When enough samples are available, further research will be needed to verify our conclusions in separate resting state functional MRI protocols. Furthermore, there is an inadequate control for age differences between tumor patients and controls, which may strongly bias gray matter results. The tumor patients have a wide range of ages from 18 to 79 years. Therefore, in order to ensure sufficient sample size, we did not adequately control for age differences. To minimize the bias of conclusion caused by age differences, we used age as a covariable to control for the effect of age differences. We are still collecting samples in succession. When enough samples are available in future, we will seriously control the age differences and then verify our conclusion in this study. Finally, we chose all subjects who were right-handed in this study, which may affect our findings. We did not evaluate whether handedness affects functional/structural reorganization according to the localization of the glioma in the dominant or in the non-dominant hemisphere. When enough left-handed samples are available in future, we will assess the effects of handedness on functional/structural reorganization.

## Conclusions

This study found that lesions invading the frontal lobe induced structural and functional reorganization of the contralesional remote areas in the lesioned area within the CCN in patients with unilateral frontal gliomas. Our findings further suggest that the contralesional superior parietal cortex acts as a functional compensation hub within the CCN, which may protect against the detrimental effects of tumor invasion on executive functions. Converging evidence provides a new perspective that avoiding damage to the compensation hub within the CCN may protect against a decline in executive function. This observation may help improve comprehensive preoperative planning for patients with frontal gliomas.

## Data Availability Statement

The datasets generated for this study are available on request to the corresponding author.

## Ethics Statement

The studies involving human participants were reviewed and approved by the responsible Human Participants Ethics Committee of the Affiliated Brain Hospital of Nanjing Medical University in Nanjing. The patients/participants provided their written informed consent to participate in this study.

## Author Contributions

YL and JC undertook the data analysis and wrote the manuscript. GH, YY, ZJ, KY, XH, ZL, DL, and YZ acquired the data. JC and HL designed the study, supervised the data analysis and provided infrastructure. All authors contributed to and have approved the final manuscript.

## Conflict of Interest

The authors declare that the research was conducted in the absence of any commercial or financial relationships that could be construed as a potential conflict of interest.
