# Supplementary material for: Structural and Functional Reorganization Within Cognitive Control Network Associated With Protection of Executive Function in Patients With Unilateral Frontal Gliomas
Source: Front Oncol. 2020 May 27;10:794. doi: 10.3389/fonc.2020.00794 (PMC7266965; doi:10.3389/fonc.2020.00794)
Supplement: Supplementary file 1 [file Data_Sheet_1.PDF]

Structural and functional reorganization within cognitive control network associated with protection of executive function in patients with unilateral frontal gliomas

Yong Liu[1, 3 #], Guanjie Hu[1,2,3 #], Yun Yu[4], Zijuan Jiang[1], Kun Yang[1,3], Xinhua Hu[1,3], Zonghong Li [3,5], Dongming Liu[1], Yuanjie Zou[1,3], Hongyi Liu[1,3*], Jiu Chen[2,3*]

[1]Department of Neurosurgery, the Affiliated Brain Hospital of Nanjing Medical University, Nanjing, Jiangsu, 210029, China

[2]Institute of Neuropsychiatry, the Affiliated Brain Hospital of Nanjing Medical University, Nanjing, Jiangsu, 210029, China

[3]Institute of Brain Functional Imaging, Nanjing Medical University, Nanjing, Jiangsu, 210029, China

[4]School of Biomedical Engineering and Informatics, Nanjing Medical University, Nanjing, Jiangsu, 211166, China

[5]Department of Radiology, the Affiliated Brain Hospital of Nanjing Medical University, Nanjing, Jiangsu, 210029, China

Running Title: Structural and functional plasticity in patients with frontal gliomas.

[#] **Yong Liu** and **Guanjie Hu** have contributed equally to this work (joint first authors).

***Correspondence to**:

Jiu Chen, Institute of neuropsychiatry, Institute of Brain Functional Imaging, the Affiliated Brain Hospital of Nanjing Medical University, No.264, Guangzhou Road, Gulou District, Nanjing, Jiangsu, 210029, China. E-mail: ericcst@aliyun.com.

Hongyi Liu, Department of Neurosurgery, the Affiliated Brain Hospital of Nanjing Medical University,

No.264, Guangzhou Road, Gulou District, Nanjing, Jiangsu, 210029, China. E-mail: hyliu18@126.com.

Supporting Information

SI methods

S.1 Image preprocessing analysis

S.2 ALFF analysis

S.3 Regional homogeneity (ReHo) analysis

S.4 Degree centrality (DC) analysis

Supplementary figure legends

Figure S1 VBM analysis comparing FronL patients with CNs

Figure S2 DC and ReHo analyses comparing FronL patients or FronR patients with CNs

Supporting Information

SI methods

S.1 Image preprocessing analysis

The imaging data were preprocessed using MATLAB2013b (http://www.mathworks.com/products/matlab/) and DPABI image processing software (Yan et al., 2016). To minimize effects of scanner signal stabilization, the first ten images were omitted from all analysis. Scans with head motion exceeding 3mm or 3° of maximum rotation through the resting-state run were discarded. After realigning, slice timing correction, and co-registration, framewise displacement (FD) was calculated for all resting state volumes (Power et al., 2012). Functional and structural images were co-registered. Structural images were then normalized and segmented into gray matter, white matter and cerebrospinal fluid signal (CSF) partitions using the DARTEL technique. The realigned fMRI data were normalized by using the EPI template into the standard MNI space and resampled to an isotropic voxel size of 3 mm, and then smoothed by a Gaussian kernel of 6 mm^3 full-width. Nuisance covarites regression including Friston 24-parameter model: 6 head motion parameters, 6 head motion parameters one time point before, and the 12 corresponding squared items (Friston et al., 1996), CSF, white matter, and the global signals as well as the linear trend were created and removed using partial regression with scrubbing (Power et al., 2014; Yan et al., 2013). After nuisance covariate regression, the resultant data were band pass filtered to select low frequency (0.01-0.1Hz) signals. Voxels within a group derived gray matter mask were used for further analyses. Finally, we also performed spatial smoothing before calculating amplitude of low-frequency fluctuations (ALFF), degree centrality (DC), and seed-based functional connectivity, and after calculating regional homogeneity (ReHo).

S.2 ALFF analysis

We used regional amplitude of low-frequency fluctuations (ALFFs) to evaluate regional functional alteration in patients. ALFFs have been consistently thought to reflect spontaneous neural activity in non-human (Shmuel and Leopold, 2008) and human (Goncalves et al., 2006). ALFF analysis procedure are described in our previously published studies (Chen et al., 2019). In brief, ALFFs of fMRI time series are calculated by measuring the magnitude of the endogenous BOLD oscillations (Y. F. Zang et al., 2007). The fMRI time series of each voxel was transformed into the frequency domain using

a fast Fourier transform, and the power spectrum was estimated. The average square root of the power spectrum was taken as the "ALFF" (Chen et al., 2019; Liu et al., 2014; Y. F. Zang et al., 2007). The ALFF maps were estimated for each voxel and standardized within each subject to generate Z-score maps (Buckner et al., 2009; Liu et al., 2014). Finally, regional estimates were calculated for each subject by averaging the Z-scores of the voxels.

S.3 Regional homogeneity (ReHo) analysis

ReHo is a measure of similarity or homogeneity of the time series in a local neighborhood of voxels (Y. Zang et al., 2004). ReHo maps were generated by calculating Kendall's coefficient concordance (KCC) of the fMRI time series of a specific voxel with all adjacent voxels (this model can be seen as a 3×3×3 cube)(Y. Zang et al., 2004). ReHo ranges from 0 to 1, with the higher values indicating greater similarity of time series in the local neighborhood. The ReHo map was then standardized by dividing the KCC of each voxel by the average KCC of the entire brain. Finally, regional estimates were calculated for each subject by averaging the Z-scores of the voxels.

S.4 Degree centrality (DC) analysis

Degree centrality (DC) represents the number of direct connections for a given voxel in the voxel-based graphs (Di Martino et al., 2013; Zuo et al., 2012). It has been widely used to represent the node characteristic of large-scale brain intrinsic connectivity networks. Specifically, the preprocessed fMRI data were used to compute Pearson's correlation coefficients between all voxel time series in the gray matter mask to obtain the DC maps (Di Martino et al., 2013; Zuo et al., 2012). Then, we constructed an n×n FC matrix of Pearson's correlation coefficients between any pair of voxels for the patient and the control, where n is the voxel number of the whole-brain mask. To obtain each subject's graph, a binary undirected adjacency matrix was formed by thresholding each correlation at $r > 0.25$, $p < 0.001$. The threshold is the default setting in the calculation of the degree centrality map and was chosen to eliminate counting voxels that had low temporal correlation attributable to signal noise. Finally, we transformed them into a Z-score matrix using Fisher's r-to-z transformation (Gao et al., 2016).

Supplementary figures and legends

Figure S1 VBM analysis comparing FronL patients with CNs

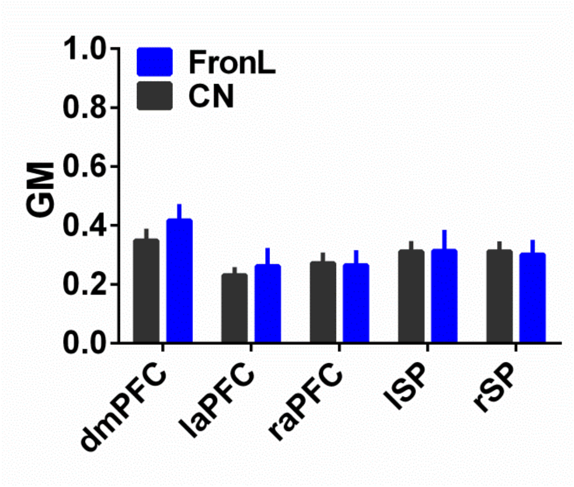

Abbreviations: DmPFC, dorsal mPFC (similar to superior medial frontal gyrus in AAL template); laPFC, left anterior PFC; raPFC, right anterior PFC; lSP, left superior parietal lobe; rSP, right superior parietal lobe; CN, controls; FrontL, patients with left frontal glioma; GM, grey matter.

Figure S2 DC and ReHo analyses comparing FronL patients or FronR patients with CNs

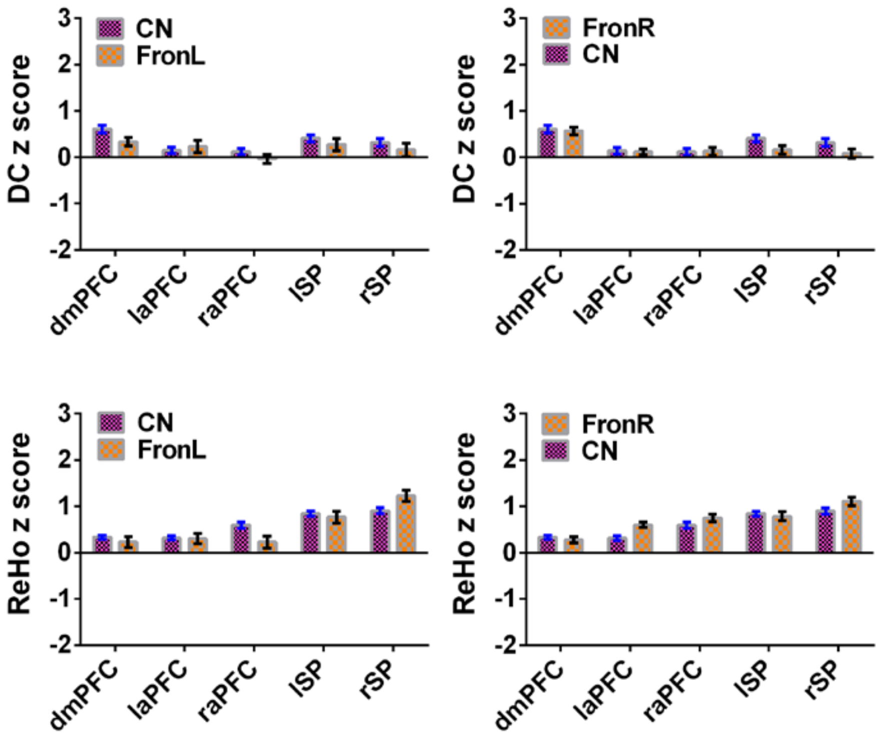

Notes: All results were thresholded at a voxel-wise $P < 0.05$ (threshold free cluster enhancement family wise error, TFCE FWE corrected) and cluster size > 30 voxels.

Abbreviations: DmPFC, dorsal mPFC (similar to superior medial frontal gyrus in AAL template); laPFC, left anterior PFC; raPFC, right anterior PFC; lSP, left superior parietal lobe; rSP, right superior parietal lobe; CN, controls; FrontL, patients with left frontal glioma; FrontR, patients with right frontal glioma; DC, degree centrality; ReHo, Regional homogeneity.

References

Buckner, R. L., Sepulcre, J., Talukdar, T., Krienen, F. M., Liu, H., Hedden, T., et al. (2009). Cortical hubs revealed by intrinsic functional connectivity: mapping, assessment of stability, and relation to Alzheimer's disease *J Neurosci* (Vol. 29, pp. 1860-1873).

Chen, J., Shu, H., Wang, Z., Zhan, Y., Liu, D., Liu, Y., et al. (2019). Intrinsic connectivity identifies the sensory-motor network as a main cross-network between remitted late-life depression- and amnestic mild cognitive impairment-targeted networks. *Brain Imaging Behav*. doi: 10.1007/s11682-019-00098-4

Di Martino, A., Zuo, X. N., Kelly, C., Grzadzinski, R., Mennes, M., Schvarcz, A., et al. (2013). Shared and distinct intrinsic functional network centrality in autism and attention-deficit/hyperactivity disorder. *Biol Psychiatry, 74*, 623-632. doi: 10.1016/j.biopsych.2013.02.011

Friston, K. J., Williams, S., Howard, R., Frackowiak, R. S., and Turner, R. (1996). Movement-related effects in fMRI time-series. *Magn Reson Med, 35*, 346-355.

Gao, C., Wenhua, L., Liu, Y., Ruan, X., Chen, X., Liu, L., et al. (2016). Decreased Subcortical and Increased Cortical Degree Centrality in a Nonclinical College Student Sample with Subclinical Depressive Symptoms: A Resting-State fMRI Study. *Front Hum Neurosci, 10*, 617. doi: 10.3389/fnhum.2016.00617

Goncalves, S. I., de Munck, J. C., Pouwels, P. J., Schoonhoven, R., Kuijer, J. P., Maurits, N. M., et al. (2006). Correlating the alpha rhythm to BOLD using simultaneous EEG/fMRI: inter-subject variability. *Neuroimage, 30*, 203-213. doi: 10.1016/j.neuroimage.2005.09.062

Liu, Y., Yu, C., Zhang, X., Liu, J., Duan, Y., Alexander-Bloch, A. F., et al. (2014). Impaired long distance functional connectivity and weighted network architecture in Alzheimer's disease. *Cereb Cortex, 24*, 1422-1435. doi: 10.1093/cercor/bhs410

Power, J. D., Barnes, K. A., Snyder, A. Z., Schlaggar, B. L., and Petersen, S. E. (2012). Spurious but systematic correlations in functional connectivity MRI networks arise from subject motion. *Neuroimage, 59*, 2142-2154. doi: 10.1016/j.neuroimage.2011.10.018

Power, J. D., Mitra, A., Laumann, T. O., Snyder, A. Z., Schlaggar, B. L., and Petersen, S. E. (2014). Methods to detect, characterize, and remove motion artifact in resting state fMRI. *Neuroimage, 84*, 320-341. doi: 10.1016/j.neuroimage.2013.08.048

Shmuel, A., and Leopold, D. A. (2008). Neuronal correlates of spontaneous fluctuations in fMRI signals in monkey visual cortex: Implications for functional connectivity at rest. *Hum Brain Mapp, 29*, 751-761. doi: 10.1002/hbm.20580

Yan, C. G., Craddock, R. C., Zuo, X. N., Zang, Y. F., and Milham, M. P. (2013). Standardizing the intrinsic brain: towards robust measurement of inter-individual variation in 1000 functional connectomes. *Neuroimage, 80*, 246-262. doi: 10.1016/j.neuroimage.2013.04.081

Yan, C. G., Wang, X. D., Zuo, X. N., and Zang, Y. F. (2016). DPABI: Data Processing & Analysis for (Resting-State) Brain Imaging. *Neuroinformatics, 14*, 339-351. doi: 10.1007/s12021-016-9299-4

Zang, Y., Jiang, T., Lu, Y., He, Y., and Tian, L. (2004). Regional homogeneity approach to fMRI data analysis. *Neuroimage, 22*, 394-400. doi: 10.1016/j.neuroimage.2003.12.030

Zang, Y. F., He, Y., Zhu, C. Z., Cao, Q. J., Sui, M. Q., Liang, M., et al. (2007). Altered baseline brain activity in children with ADHD revealed by resting-state functional MRI. *Brain Dev, 29*, 83-91. doi: 10.1016/j.braindev.2006.07.002

Zuo, X. N., Ehmke, R., Mennes, M., Imperati, D., Castellanos, F. X., Sporns, O., et al. (2012). Network centrality in the human functional connectome. *Cereb Cortex, 22*, 1862-1875. doi: 10.1093/cercor/bhr269
